# Toll-Like Receptor 4, but Not Neutrophil Extracellular Traps, Promote IFN Type I Expression to Enhance Th2 Responses to *Nippostrongylus brasiliensis*

**DOI:** 10.3389/fimmu.2017.01575

**Published:** 2017-11-16

**Authors:** Christophe Pellefigues, Shiau-Choot Tang, Alfonso Schmidt, Ruby F. White, Olivier Lamiable, Lisa M. Connor, Christiane Ruedl, Jurek Dobrucki, Graham Le Gros, Franca Ronchese

**Affiliations:** ^1^Malaghan Institute of Medical Research, Wellington, New Zealand; ^2^School of Biological Sciences, Nanyang Technological University, Singapore, Singapore; ^3^Faculty of Biochemistry, Biophysics and Biotechnology, Department of Cell Biophysics, Jagiellonian University, Kraków, Poland

**Keywords:** *Nippostrongylus brasiliensis*, helminth, dendritic cells, toll-like receptor 4, IFN-I, neutrophil extracellular traps, Th2 response, skin immunity

## Abstract

The induction of Th2 responses is thought to be multifactorial, and emerge from specific pathways distinct from those associated with antagonistic antibacterial or antiviral Th1 responses. Here, we show that the recognition of non-viable *Nippostrongylus brasiliensis* (Nb) in the skin induces a strong recruitment of monocytes and neutrophils and the release of neutrophil extracellular traps (NETs). Nb also activates toll-like receptor 4 (TLR4) signaling with expression of *Ifnb* transcripts in the skin and the development of an IFN type I signature on helminth antigen-bearing dendritic cells in draining lymph nodes. Co-injection of Nb together with about 10,000 Gram-negative bacteria amplified this TLR4-dependent but NET-independent IFN type I response and enhanced the development of Th2 responses. Thus, a limited activation of antibacterial signaling pathways is able to boost antihelminthic responses, suggesting a role for bacterial sensing in the optimal induction of Th2 immunity.

## Introduction

Diverse immune responses have been associated with different classes of pathogen or insult, and with the specialization of CD4^+^ T helper cells toward the secretion of a certain set of effector cytokines: Th1 cells secreting Interferon-γ (IFNγ) are generated in antitumoral, antibacterial, and anti-“intracellular pathogen” responses, Th2 cells secreting interleukin 4 (IL4), IL5, and/or IL13 are generated in anti-venom/toxin/irritant and anti-macroparasite (including helminths and ticks) responses, and Th17/22 cells secreting IL17 or IL22 are generated upon antifungal and antibacterial responses (1–3).

Tissue resident dendritic cells (DCs) are critical for the priming of antigen specific T cell responses. They are able to shape the polarization of adaptive immunity toward a Th1, Th2, or Th17 response by interpreting signals from their environment (1). While the sensing of viral and bacterial nucleic acids by intracellular Pattern Recognition Receptors, and of bacterial lipopolysaccharides (LPS) by Toll-like receptor 4 (TLR4) have been shown to induce the development of strong Th1 responses, a consensus signal required to induce the development of Th2 immune responses has not been identified. Several types of signals can participate in inducing a Th2 response including, but not limited to, tissue derived cytokines and alarmins such as TSLP, IL25, and IL33, an innate “third party” source of IL4, the activity of specific proteases or phospholipases, some macroparasite glycans or glycolipid motifs, and the detection of extremely low amounts of LPS (1, 3–7). Understanding the signals that govern the development of Th2 responses is of uttermost importance considering that helminths potentially affect around 25% of the world population (8, 9) while allergic diseases can affect more than 30% of the population (10), mainly in areas where helminthiases are not endemic.

We unexpectedly identified that the development of Th2 responses to the prototypical helminth *Nippostrongylus brasiliensis* (Nb) is partly dependent on IFN type I (IFN-I) signaling (11). It has also been shown very recently that IFN-I is important for the initiation of Th2 responses to *Schistosoma mansoni* and House dust mite (HDM) by DCs (12). Indeed, 2 days after the injection of non-viable AF488-labeled Nb into the ear dermis, AF488^+^ DCs infiltrating the draining lymph nodes (dLNs) showed a strong transcriptomic IFN-I signature. These AF488^+^ DCs also showed a phenotypic IFN-I signature as demonstrated by their expression of several IFN-I-dependent cell surface markers including bone marrow (BM) stromal antigen 2 (BST2, or CD317). Neutralizing IFN-I signaling with αIFNAR1 antibodies was able to diminish the expansion of IL4-secreting CD4^+^ T cells in response to Nb (11). As IFN-I signaling is generally associated with the development of antiviral, antibacterial, or autoimmune responses (13–15), and with DC activation and maturation (16–21), we sought to identify by which mechanisms Nb could induce an IFN-I response.

The release of endogenous oxidized DNA during cell death or neutrophil extracellular traps (NETs) secretion is strongly immunogenic and interferogenic in various pathophysiological contexts (22–25). As neutrophils are important for the immune response to Nb (26–28), we investigated whether Nb injection induced the secretion of NETs in the skin. To this end, we used a very controlled and defined system utilizing non-viable L3 Nb larvae injected into the ear dermis (29). This enabled us to monitor the development of the immune response in local tissue and auricular dLNs in the absence of potential interfering factors such as local tissue damage or infection-related systemic effects.

We show that the injection of non-viable L3 Nb larvae into the ear dermis induces recruitment of neutrophils undergoing NETosis around the worms. Surprisingly, NET digestion or depletion of neutrophils were not sufficient to diminish the IFN-I signature on AF488^+^ DCs. Interestingly, expression of IFN-I in the skin, and the IFN-I-dependent upregulation of BST2 on dLN DCs, required expression of TLR4. Consistent with this observation, adding Gram-negative bacteria to AF488^+^ Nb before injection increased the expression of BST2 on AF488^+^ DCs in dLN, and the magnitude of the resulting Th2 response. These findings strongly suggest that metazoan parasite TLR4 ligands, originating from their associated microorganisms and/or also from their cuticle glycans (7, 30), induce the secretion of IFN-I to enhance DC maturation and the development of specific Th2 responses.

## Materials and Methods

### Mice and Treatments

Seven- to 10-week-old female C57BL/6J, SiglecH-DTR (31), TLR2 KO (32), and TLR4 KO (32) mice were bred and housed in specific pathogen-free conditions at the Malaghan Institute of Medical Research Biomedical Research Unit. All experimental protocols were approved by the Victoria University of Wellington Animal Ethics Committee (Permit 2014R17M) and performed according to Institutional guidelines.

*Nippostrongylus brasiliensis* infective L3 larvae (Nb) were collected, washed in sterile PBS, killed by three freeze–thaw cycles, and injected intradermally (i.d.) into the ear pinna of anesthetized mice as previously described (29). “Low Endotoxin” Nb preparations (LE-Nb) were achieved by adding five extra washing steps to the preparation. Endotoxin content was quantified using the LAL Chromogenic Endotoxin quantitation kit (Pierce) and was <5 EU/mL. Nb sterilization was achieved by antibiotic treatment as previously described (27). In some experiments, Nb was labeled using Alexa Fluor 488 (AF488) succinimidyl ester dyes (Molecular Probes) as described previously (11). To prepare Nb supernatant (SN), Nb suspensions were left to sediment for 5 min at room temperature (RT), and SN collected.

To block IFN-I signaling *in vivo*, mice were treated i.d. with 250 µg MAR1-5A3 (blocking anti-mouse IFN-alpha and beta receptor 1 antibody, anti-IFNAR1) or isotype control (MOPC-21) given with Nb on day 0. The same antibody dose was given again on day 2 by intraperitoneal (i.p.) injection. “*In Vivo* Plus” MAR1-5A3 and MOPC-21 were from BioXCell (West Lebanon, NH, USA).

To deplete plasmacytoid DCs (pDCs), SiglecH-DTR mice were given 25 ng/g diphtheria toxin (DT, Sigma) i.p. 1 day before Nb injection. In all experiments, flow cytometry analysis of spleen cells confirmed >95% depletion of pDCs, identified as CD11b^−^ CD11c^+^ B220^+^ Ly6C^+^ BST2^+^ cells, compared to DT-untreated controls. Neutrophils were depleted by injecting 0.5 mg anti-Ly6G antibody or 200 µg anti-Gr1 antibody versus the same amount of their isotype control (IA8 or RB6-8C5, respectively, InVivoPlus, BioXCell) i.p. 1 day before and on the day of Nb injection. Depletion was assessed in skin by enumerating neutrophil infiltration as CD45^+^ CD11b^+^ Ly6C^int^ Ly6B^+^ cells (50–70% depletion) or CD45^+^ CD11b^+^ Ly6G^+^ cells (>95% depletion). To digest NETs, mice were injected with 2,000 U DNase I i.d. (Roche) together with Nb, followed by 2,000 U i.p. every 12 h until the end of the experiment. NET digestion was qualitatively confirmed by microscopy at 2 h after DNase I injection (33, 34).

### Quantitative Reverse Transcription PCR (RT-qPCR)

Ears were collected at the indicated times and stored in RNALater (Invitrogen) at 4°C. Tissue was cut into small pieces with scissors and homogenized using TissueLizer II (Qiagen) and RNA was extracted with Trizol (Invitrogen) following the supplier’s instructions. cDNA was synthetized using the High capacity RNA-to-cDNA kit (Applied Biosystems). RT-qPCR was performed using SYBR Green Master Mix and the following primers: Beta Actin (F:5′ CTAAGGCCAACCGTGAAAAG, R:5′ ACCAGAGGCATACAGGGACA), Ifnk (F:5′ CCGCCCATCCAATCTCTGAA, R:5′ GGAAAGCCGGTCATGGTACT), Ifnb1 (F:5′-GCACTGGGTGGAATGAGACT, R: 5′-AGTGGAGAGCAGTTGAGGACA), and Ifna (F: 5′-TCTGATGCAGCAGGTGGG, R: 5′-AGGGCTCTCCAGACTTCTGCTCTG) to amplify all Ifna species (16), using a QuantStudio 7 (Applied Biosystems) and following the manufacturer’s guidelines. Transcript levels are expressed as the ratio of 2^−ΔCT^ (Transcript of interest)/2^−ΔCT^ (Beta Actin) and normalized by comparison to pertinent experimental controls.

### Cell preparations and Flow Cytometry

For DC preparations, auricular dLNs were harvested and digested for 30 min at 37°C in IMDM (Gibco) containing 100 µg/mL DNase I and 100 µg/mL Liberase TL (Roche). For T cell preparations, LNs were passed through a 70-µm cell strainer (Falcon). For skin cell preparations, ears were split into the dorsal and ventral layers, and then minced in Accutase (Stemcell) containing 3 U/mL Dispase II, 100 µg/mL DNAse I, and 100 ng/mL Liberase TM (Roche) for 30 min at 37°C.

Peripheral blood leukocytes were harvested by cheek puncture and red blood cells were lysed in an ammonium chloride/TRIS buffer, as detailed elsewhere (35). Single cell suspensions were filtered on 70-µm nylon mesh cell strainers (Falcon) and blocked for 15 min at 4°C in FACS Buffer (PBS 1% bovine serum albumin 0.05% NaN_3_) containing anti-mouse CD16/CD32 from affinity purified 2.4G2 hybridoma SN. Cells were then stained in FACS Buffer for 20 min at 4°C with an optimized concentration of fluorophore-conjugated antibodies.

For intracellular cytokine staining, cells were cultured in complete IMDM containing 10% Fetal Calf Serum (FCS) and penicillin/streptomycin (all from Gibco) and stimulated with Phorbol 12-Myristate 13-Acetate (50 ng/mL) and Ionomycin (1 µg/mL) for 5 h at 37°C in the presence of GolgiStop (BD Bioscience). After surface staining, cells were fixed and permeabilized with the Cytofix/Cytoperm kit (BD Bioscience) and stained intracellularly.

The fluorescent antibodies used were specific for CD11c (HL3), CD86 (GL1), MHCII (M5/114), CD326 (G8.8), CD4 (RM4-5), CD3 (145-2C11), CD103 (M290), IL4 (11B11), IFNg (XMG1.2), and CD44 (IM7; all from BD); IL10 (JES5-16E3), CD8a (53-6.7), CD11b (M1/70), CD45 (30-F11), CD64 (X54-5/7.1FC), Ly6C (HK1.4), Ly6G (IA8), CD317 (BST2, clone 927) from BioLegend; IL17A (eBio17B7) and B220 (RA3-6B2; both from eBioscience); Ly6B.2 (7/4) from Thermofischer. IL4-AmCyan expression was quantified with a 504/12 filter after excitation at 445 nm. Non-viable cells and doublets were identified and excluded using DAPI or LIVE/DEAD staining (Molecular Probes). Compensations were performed using OneComp eBeads (Invitrogen) as single stained positive controls and fluorescence minus one (FMO) controls were used to set background expression. Flow cytometry was performed on a BD LSRII or LSR Fortessa SORP flow cytometer with FacsDiva 6.1.1 software (Becton Dickinson). Analyses were conducted using FlowJo vX (Tree Star) and the represented values of expression intensity are the geometric means of fluorescence intensity (mfi).

### Cell Culture

Human Embryonic Kidney 293 (HEK) cell lines engineered to report NF-κB activation with the secretion of an optimized alkaline phosphatase were used to study the signaling pathways activated by Nb. HEK-Blue cells expressing murine TLR2, TLR4, TLR7, or TLR9, and their respective control cell lines (Null1, Null2, Null1-v, Null2-k) were cultured in triplicate in 96 well plates at 50,000/well, and stimulated O/N at 37°C with either 100 Nb or the appropriate positive controls. Reporter expression was assessed using QuantiBlue medium (InvivoGen) following manufacturer’s instructions. Absorbance at 640 nm was measured on a spectrophotometer (Helios Gamma, ThermoScientific). Data from each reporter cell line were expressed as ratio of the means of stimulated versus unstimulated cultures.

Primary BM cells were harvested by flushing femurs’ content using medium. One million cells were cultured in RPMI and 10% FCS (Gibco) for 24 h at 37°C, in the presence of 10 µg/mL InVivoPlus mouse IgG1 (MOPC1) or anti-IFNAR1 (MAR-5A3) from Bio X Cell (Lebanon, NH, USA). Cultures were stimulated with HDM whole bodies (Greer) at 100 µg/mL, or 10–200 Nb, or 10 µg/mL low-endotoxin Poly(I:C) (Invitrogen). *Escherichia coli* (*E. coli*) MG1655 was grown in Luria Broth (Invitrogen) at 37°C, quantified by OD, harvested at exponential growth phase, and fixed in 1% formalin.

### Microscopy

One hundred and fifty AF488-labeled Nb were injected into the ear dermis in 30 µl of sterile PBS. After different times, mice were euthanized and hair was removed with a hair removal cream (Veet) before harvesting the ears. The ear dorsal and ventral parts were split and fixed in 4% formalin (Sigma) for 30 min, washed in PBS, permeabilized and blocked in PBS with 0.3% Triton X100 (PBS-T) 5% Donkey serum and 2.4G2 hybridoma SN at RT for 30 min. The same buffer was used for antibody staining: primary antibody staining was performed using 5 µg/mL of goat anti human/mouse MPO (R&D, AF3667), 1/500 rabbit anti-Histone H3 citrulline (R2 + R8 + R17, ab5103) or 1/100 rabbit anti-mouse Neutrophil elastase (ab21595, both from Abcam) for 2 h at RT or overnight at 4°C. Samples were washed five times in PBS-T and then stained for 1 h using 1/500 donkey anti-goat AF594 (ab140150) and/or 1/500 donkey anti-rabbit AF647 (ab181347, both from Abcam). After five washes, tissue was mounted under a coverslip in Fluoromount (Sigma) and recorded using a Confocal Laser Scanning microscope FV1200-IX83 (Olympus). Image analysis and tri-dimensional reconstructions were done with the Fiji version of ImageJ and the help of a 3D viewer plugin to juxtapose z-stacks (36, 37).

### Statistical Analysis

Statistical analyses were performed using Prism 7.0 (GraphPad). The distribution of the data groups was always assessed using a Shapiro–Wilk test for normality. Data groups were compared using one way ANOVA with a Tukey’s multiple comparison test for experiments following a normal distribution, or using the Mann–Whitney tests, or, when more than three independent groups were considered, a Kruskal–Wallis with a Dunn’s multiple comparison test for experiments whose data points were not following a normal distribution. In all cases, a two-tailed *p* value < 0.05 was considered as threshold for significance. Mean and SEM are shown in all graphs.

## Results

### The Majority of Cells Taking up Nb Material in the Skin at 24 h Are Neutrophils and Inflammatory Monocytes

**Figure 1:**
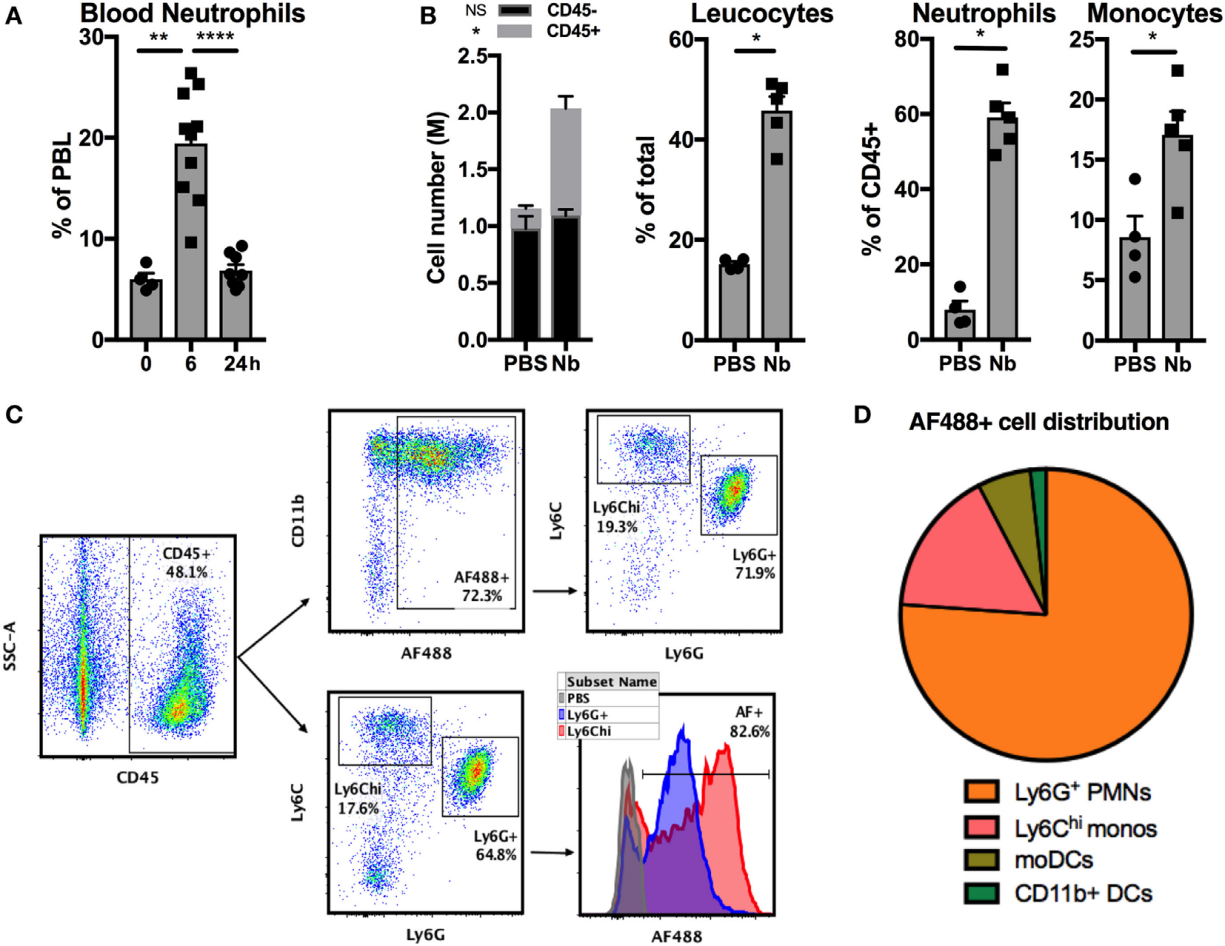
*Nippostrongylus brasiliensis* (Nb) induces the recruitment and activation of neutrophils in the skin. C57BL/6 mice were injected intradermally into the ear with 600 AF488^+^ Nb or PBS. The resulting inflammatory response in blood and skin was analyzed by flow cytometry at the indicated time points. Each symbol represents one mouse. **(A)** Proportion of neutrophils (CD11b^hi^ Ly6G^+^) in peripheral blood leukocytes (PBL). Data are pooled from two independent experiments. **(B)** Recruitment of leukocyte populations in the ear skin at 24 h. Leukocytes were identified as CD45^+^ cells, neutrophils and monocytes were identified according to the gating in panel C. Data are from one of three experiments that gave similar results. **(C)** Representative dot plots depicting the gating of ear skin CD45^+^ populations expressing Ly6G (neutrophils), high Ly6C (monocytes), and AF488 as a measure of Nb uptake. **(D)** Pie chart showing the relative proportion of AF488^+^ populations in skin, identified as in **(C)**. Monocyte-derived DCs (moDCs) are defined as Ly6G^−^ Ly6C^hi^ CD11b^hi^ CD11c^+^ MHCII^+^, and CD11b^+^ dendritic cells (DCs) are Ly6G^−^ Ly6C^−^ CD11c^+^ MHCII^+^ CD11b^+^ CD326^−^. Data are from one of three experiments that gave similar results. Bar graphs show mean ± SEM. Statistical analyses used the Mann–Whitney test. NS: not significant; *p < 0.05; **p < 0.01; ****p < 0.0001. **(B)** Symbols close to the legend indicate a comparison of the same population between the two groups.

Stage 3 (L3) larvae from the helminth Nb can infect rodents by penetrating their skin barrier. To study the development of antihelminth Th2 responses in skin we used a simplified model involving injection of non-viable Nb into the ear dermis of mice (29, 38). Nb injection induced a quick and transient neutrophilia in the blood (Figure 1A) and an accumulation of CD45^+^ leukocytes in the ear dermis comprising mainly CD11b^hi^ Ly6G^+^ neutrophils and CD11b^hi^ Ly6G^−^ Ly6C^hi^ inflammatory monocytes at 24 h (Figure 1B).

The reactive amines in the cuticle of Nb larvae can be stained covalently with the succinimidyl ester AF488, to generate AF488^+^ Nb larvae (11, 38). 24 h after AF488^+^ Nb injection, approximately 90% of the cells that had taken up AF488 in the dermis were neutrophils and inflammatory monocytes. Indeed, the majority of these two cell types stained positively for AF488 (Figures 1C,D). Of note, AF488 fluorescence was consistently brighter in monocytes than neutrophils, which might be due to different phagocytosis rates, dye stability, or apoptosis of these cell populations.

These observations show that neutrophils and inflammatory monocytes are recruited early to the ear dermis to interact with non-viable Nb.

### Injection of Non-viable Nb Induces NETosis in the Dermis

**Figure 2:**
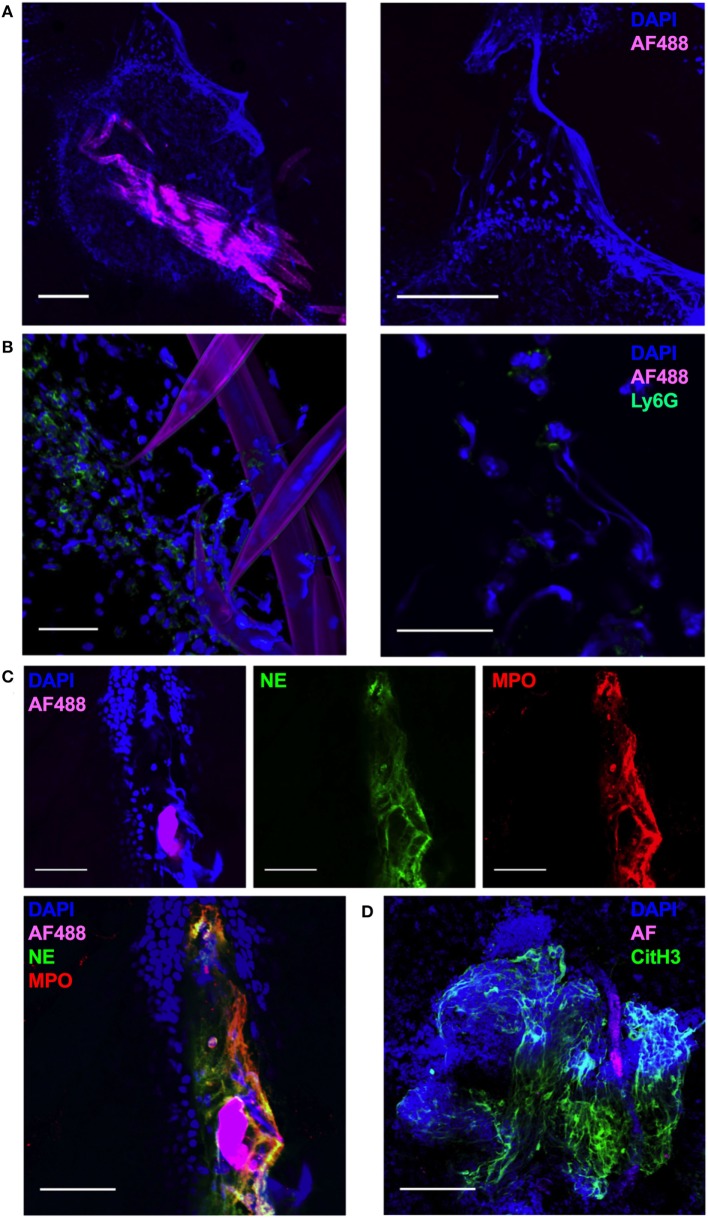
*Nippostrongylus brasiliensis* (Nb) induces the formation of neutrophil extracellular traps in skin. C57BL/6 mice were injected intradermally into the ear with 150 AF488^+^ Nb, and z-stacks of whole-mount ear dermis were analyzed by immunofluorescence and confocal microscopy at different time points. Images are representative of at least two independent experiments. **(A)** Dermis at 1 h after AF488^+^ Nb injection showing DAPI^+^ nuclei (blue) and AF488^+^ Nb (magenta). Bar = 200 µm. **(B)** Dermis at 2 h after AF488^+^ Nb injection showing DAPI^+^ nuclei (blue), AF488^+^ Nb (magenta), and Ly6G^+^ neutrophils (Green). Bar = 50 µm. **(C)** Dermis at 24 h after AF488^+^ Nb injection showing DAPI^+^ nuclei (blue), AF488^+^ Nb (magenta), myeloperoxidase (Red), and neutrophil elastase (Green). Bar = 50 µm. **(D)** Dermis at 48 h after AF488^+^ Nb injection showing DAPI^+^ nuclei (blue), autofluorescent Nb (magenta), histone H3 citrullination (Green), and neutrophil elastase (Red). Bar = 200 µm.

In order to visualize the early events in the interaction of non-viable Nb with infiltrating leukocytes, we carried out a whole-mount confocal imaging of fixed ear dermis. An increased density of DAPI^+^ cells close to the injected Nb was observed as early as 1 h post-injection (p.i.) (Figure 2A, left). Surprisingly, in addition to the cellular infiltrate, worms were also surrounded by large DAPI^+^ fiber-shaped structures (Figure 2A, right). By 2 h after Nb injection, most of the cells associated with Nb were Ly6G^+^ neutrophils (Figure 2B, left). Interestingly, the Ly6G staining was often associated with the formation of extracellular DAPI^+^ fiber-shaped structures, suggesting a neutrophil mediated phenomenon induced less than 1 h after Nb injection (Figure 2B, right).

Immunohistochemistry experiments to investigate the nature of the DAPI^+^ structures surrounding Nb showed that extracellular DNA fibers were often co-localized in the extracellular space with the neutrophil granule enzymes neutrophil elastase (NE, Green) and myeloperoxidase (MPO, Red) (Figure 2C), commonly found in NETs. NET formation has been shown to be dependent on peptidyl arginine deiminase 4 activity and its histone H3 citrullination. Peptidyl arginine deiminase 4 facilitates nuclear DNA decondensation, and the release into the extracellular space of DNA coated by neutrophil granule enzymes and histones. Indeed, extracellular DNA fibers could be observed in the dermis for at least 48 h after Nb injection, and stained strongly for histone H3 citrullines thus identifying them as NETs (Figure 2D) (39–42).

### *Nippostrongylus brasiliensis* Induces an IFNAR1-Dependent and TLR4-Dependent Expression of BST2 on BM Cells

**Figure 3:**
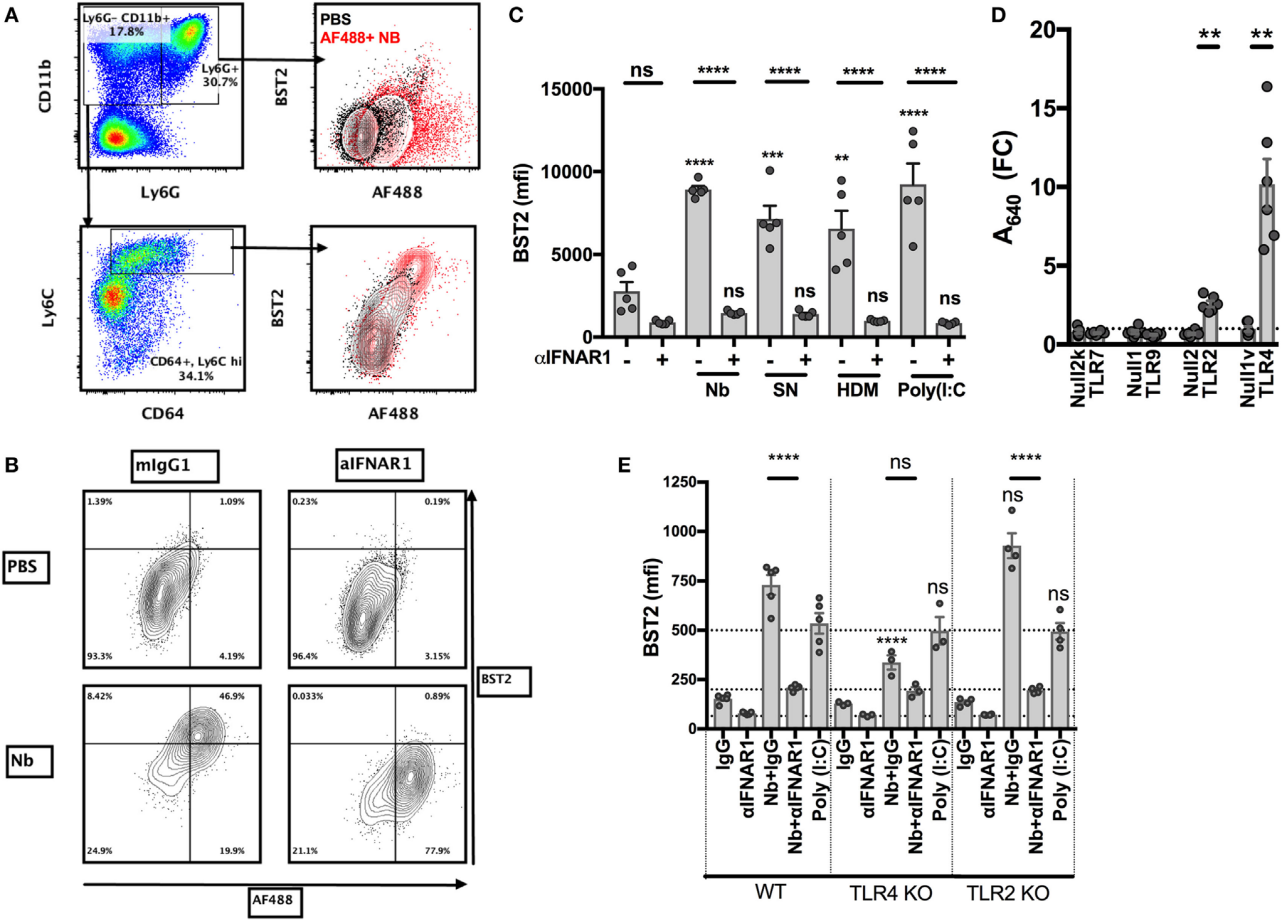
Toll-like receptor 4 (TLR4) expression is necessary for *Nippostrongylus brasiliensis* (Nb)-dependent BST2 expression *in vitro*. Bone marrow (BM) cells were harvested from C57BL/6 mice or the indicated strains, stimulated overnight in the described conditions, and analyzed by flow cytometry. **(A)** Identification of neutrophils (CD11b^hi^ Ly6G^+^) and monocytes (CD11b^hi^ Ly6G^−^ Ly6C^+^) and their uptake of AF488 and expression of BST2 after stimulation with AF488^+^ Nb (red) or medium (black). **(B)** BST2 expression on monocytes from BM cultures that were stimulated with AF488^+^ Nb in the presence of IFNAR1-blocking antibodies or isotype control. **(C)** BST2 expression on monocytes from BM cultures stimulated with Nb, Nb supernatant (SN), house dust mite (HDM) or Poly(I:C) in the presence of IFNAR1-blocking antibodies or mIgG1. Data are from one of at least two independent experiments that gave similar results; each dot corresponds to a BM culture from a separate mouse. **(D)** Stimulation of TLR reporter activity by Nb. Cell lines expressing the indicated TLR and their respective controls were cocultured overnight with Nb or no stimulus, and TLR reporter activity was quantified using a colorimetric assay. Reporter activity for each cell line is expressed as fold-change (FC) of the readings for stimulated versus unstimulated cultures. Each dot corresponds to an independent experiment and is the average of triplicate cultures. **(E)** BM cells from C57BL/6 (WT), TLR4 KO or TLR2 KO mice were cultured with Nb or Poly(I:C) or no stimulus, in the presence of IFNAR1-blocking antibodies or isotype control. BST2 expression on monocytes was assessed after overnight culture. Data are from one of at least two independent experiments that gave similar results; each dot corresponds to a BM culture from a separate mouse. Statistical analyses used the ANOVA with Tukey’s multiple comparisons test. NS: not significant; **p < 0.01, ***p < 0.001, ****p < 0.0001. Symbols above individual bars refer to the *p* value of the indicated group versus its control condition, which was either unstimulated **(C)** or WT **(E)**.

We recently showed that the injection of non-viable Nb in the ear dermis induces a strong IFN-I transcriptional signature on the migratory DCs infiltrating the skin dLN, and that IFN-I signaling is important for an optimal Th2 immune response in this model (11). In order to study the Nb sensing mechanisms that lead to IFN-I secretion, we used primary BM cells as they contain high proportions of neutrophils and monocytes, which are the main cell types recruited to the ear dermis early after Nb injection. An analysis of the surface markers expressed by these cell populations showed that, compared to AF488^−/low^ cells, AF488^+^ inflammatory monocytes, and to a lesser extent neutrophils, overexpressed the IFN-I-induced marker BST2 (11, 43) (Figure 3A). Treatment with IFNAR1-blocking antibodies reversed BST2 upregulation, confirming that it was dependent on IFNAR1 signaling (Figures 3B,C). Coculture with SN from Nb preparations, to determine if the IFN-I inducing factor present in Nb preparations sedimented with the Nb body or was a soluble factor, and coculture with house dust mite whole bodies (HDM) also induced an IFN-I-dependent upregulation of BST2 (Figure 3C). This latter observation is consistent with a very recent and elegant report showing that *Schistosoma mansoni* and HDM induce an IFN-I dependent Th2 response (12). Thus, several Th2 stimuli can induce the secretion of IFN-I by primary BM cells.

IFN-I is known to be produced in high amounts during antiviral and cellular immune responses, but much less is known about its secretion during metazoan parasite infections or other Th2 responses (12, 44). The mechanisms leading to the secretion of IFN-I have been mostly associated with the detection of viral nucleic acids or bacterial LPS (45–47). We used commercial toll-like receptor (TLR) reporter cell lines to investigate whether Nb preparations were able to trigger active signaling through these receptors. *In vitro*, non-viable Nb larvae induced the activation of NF-κB only in reporter cell lines expressing TLR2 or TLR4, but not in those expressing TLR7 or TLR9, or their respective controls (Figure 3D). This suggests that Nb-induced IFN-I secretion is unlikely to be mediated by nucleic acid sensing, and that Nb can instead be detected by TLR2 and TLR4.

Coculture of non-viable Nb with fresh BM cells showed that Nb could induce an IFNAR1-dependent expression of BST2 on WT and TLR2 KO, but not TLR4 KO, BM monocytes (Figure 3E; Figure S1A in Supplementary Material), which is consistent with the reported differential capacity of these receptors to trigger IFN-I secretion (47). Anti-IFNAR1 antibody treatment decreased BST2 expression below control levels, suggesting a constitutive IFN-I signaling in BM cultures. Of note, IFNAR1 expression on monocytes was decreased upon Nb stimulation of WT and TLR2 KO BM, but was unaffected in the TLR4 KO BM cultures (Figure S1B in Supplementary Material). This is consistent with a ligand-induced endocytosis of IFNAR1, and an absence of IFN-I secretion only in the TLR4 KO background. Importantly, IFNAR1 expression and Nb-AF488 dye uptake were not influenced by TLR2 or TLR4 expression, revealing that these primary cells were not defective in IFN-I secretion, signaling, or phagocytosis in these conditions (Figure 3E; Figure S1C in Supplementary Material).

BST2 expression on monocytes and neutrophils was dose-dependent and saturable after induction by Nb or by formalin-fixed Gram-negative *E. coli* bacteria (data not shown). These experiments confirmed that the secretion of IFN-I induced by Gram-negative bacteria, known to be dependent on TLR4 signaling (47), could also be observed in BM cultures.

These results show that Nb preparations are able to signal through TLR2 and TLR4 in BM cells, and elicit the upregulation of BST2 expression *via* a TLR4-dependent and IFNAR1-dependent pathway. However, the precise nature of these TLR4 ligands remains to be defined.

### TLR4 Mediates Nb-Induced *Ifnb* Expression, but Not NETosis, in the Skin

**Figure 4:**
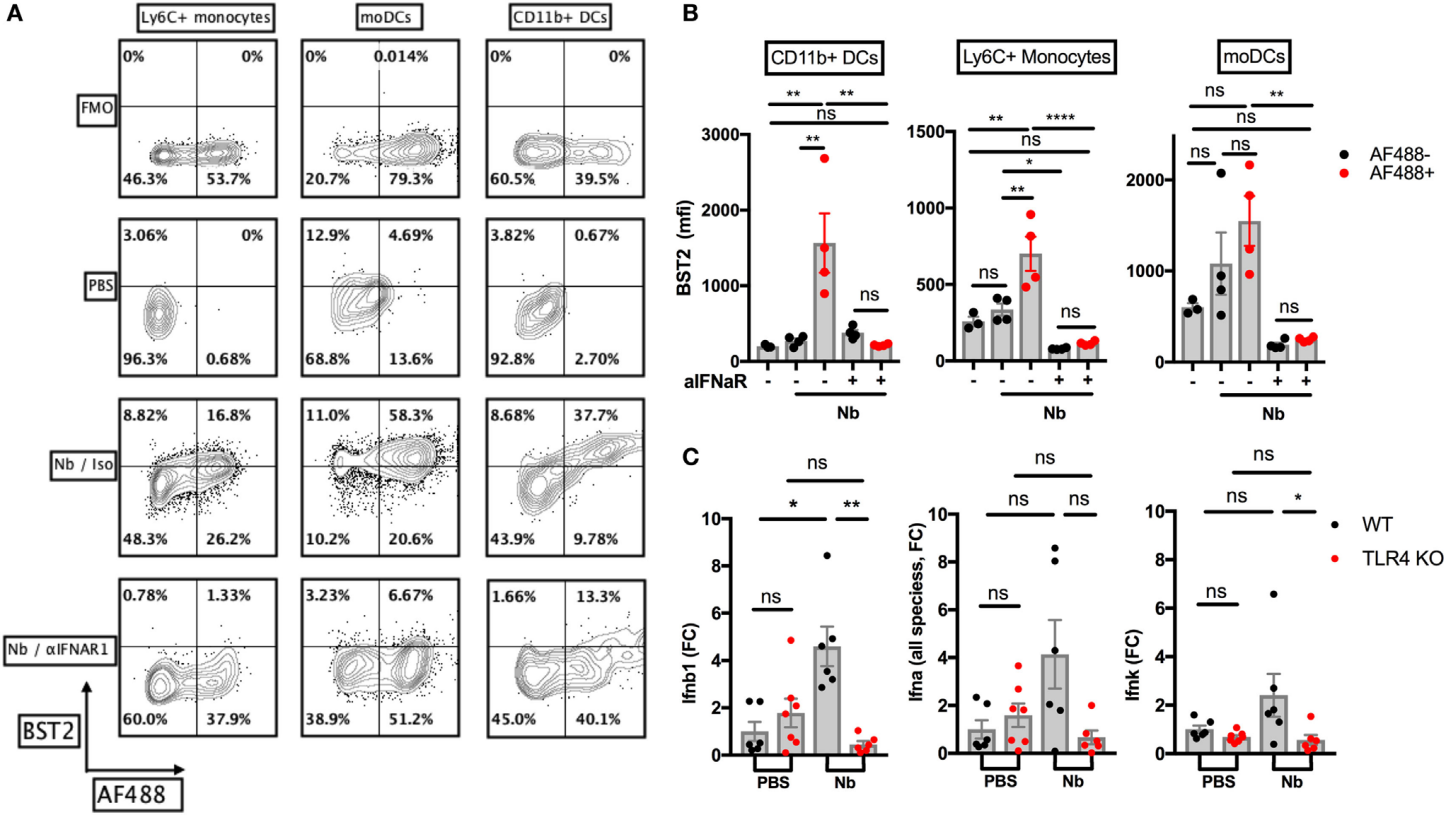
*Nippostrongylus brasiliensis* (Nb) induces a toll-like receptor 4 (TLR4)-dependent expression of IFN-I in the skin. C57BL/6 (WT) or TLR4 KO mice were injected intradermally into the ear with 600 AF488^+^ Nb. Expression of IFN-I transcripts and of the IFN-I-dependent marker BST2 were examined in ear tissue as indicated. Each dot corresponds to one mouse. **(A)** Representative flow cytometry contour plots depicting AF488 uptake and expression of BST2 on CD45^+^ skin populations 24 h after PBS injection, or co-injection of AF488^+^ Nb together with IFNAR1-blocking antibodies or isotype control. Cell populations were identified as in Figure 1. Fluorescence Minus One (FMO) for BST2 is shown as a control. **(B)** Expression of BST2 on AF488^−^ and AF488^+^ skin populations defined as in **(A)**. Bar graphs show mean ± SEM from one of two independent experiments that gave similar results. **(C)** Quantitative Reverse Transcription PCR for *Ifnb1, Ifna* (all species) and *Ifnk* in ear tissue 2 h after Nb injection. Bar graphs show mean ± SEM from two independent experiments each with three mice/group. Statistical analyses used the ANOVA with Tukey’s multiple comparisons test in **(B)**, or Kruskal–Wallis test with Dunn’s multiple comparison in **(C)** as the distribution of the data could not be considered normal (Shapiro–Wilk normality test). Ns: not significant; *p < 0.05; **p < 0.01.

We wished to assess the expression of IFN-I in the skin after Nb injection. Non-viable AF488^+^ Nb larvae induced expression of the IFN-I-induced marker BST2 on AF488^+^ monocytes, monocyte-derived DCs and CD11b^+^ dermal DCs in the skin at 24 h p.i. (Figures 4A,B), implying that IFN-I was secreted in the skin before 24 h. The main components of the IFN-I family are transcribed from 14 *Ifna*, 1 *Ifnb*, 1 *Ifnk*, and 1 *Ifne* genes (48). Using RT-qPCR, we detected a transient expression of *Ifnb1* in ear skin, peaking at 2 h p.i. and disappearing quickly at later time points (Figure S2A in Supplementary Material). Other common IFN-I species including *Ifna2* and *Ifna4* were not detected in significant amounts during the first 6 h p.i. (data not shown). When Nb-induced *Ifn* expression was assessed in C57BL/6 and TLR4 KO mice, no *Ifnb1* transcripts could be detected in TLR4 KO mice. Of note, we could also observe a trend toward a low expression of *Ifna* (all species) (16) and *Ifnk* in C57BL/6 recipients, but again this was not observed in TLR4 KO mice (Figure 4C).

Plasmacytoid DCs are known to be the main producer of IFN-I on a per cell basis in antiviral responses and some autoimmune diseases. In these conditions, pDC IFN-I secretion is mainly induced by the detection of nucleic acids through TLR7 and TLR9, whereas the TLR4 pathway is not involved (49). We, therefore, used SiglecH-DTR mice to assess the contribution of pDCs to IFN-I production after Nb injection. Depletion of pDCs by i.p. treatment with DT had no effect on the expression of *Ifnb1* in ear skin 2 h after Nb injection (Figure S2B in Supplementary Material). Transcripts for *Ifna* or *Ifnk*, which could be detected at low levels in C57BL/6 mice, were undetectable in SiglecH-DTR mice on a Balb/c background (Figure S2B in Supplementary Material). These discrepancies might be explained by cell type and strain-specific differences in the expression of IFN-I family members (50). Together, these results suggest that pDCs are not involved in the IFN-I response to Nb.

NETosis is known to be induced by various signaling pathways, including TLR2 and TLR4 activation (51, 52). As NETosis has been associated with the secretion of IFN-I by various cell types (22–24, 53), we investigated whether Nb-induced NETosis required the expression of TLR2 or TLR4. We were able to observe NETosis in the skin of mutant mice as early as 1 h after non-viable Nb injection, ruling out a key requirement for either TLR2 or TLR4 in mediating Nb-induced NETosis (Figure S3 in Supplementary Material).

### Expression of an IFN-I signature on migratory DC requires host TLR4 expression but not NET formation

**Figure 5:**
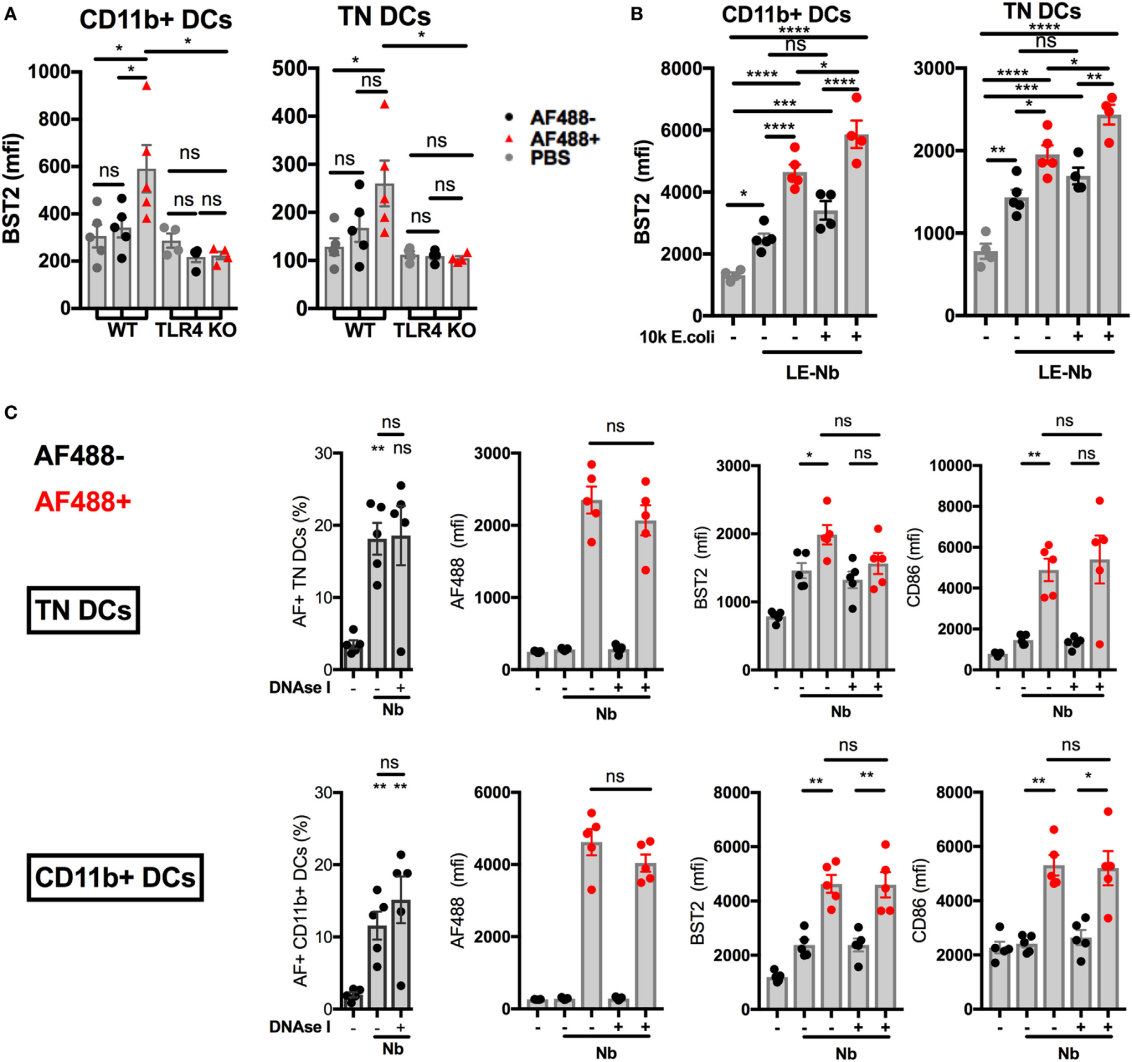
Toll-like receptor 4 (TLR4), not extracellular DNA, drives the development of an IFN-I signature on LN dendritic cells (DCs) after *Nippostrongylus brasiliensis* (Nb) injection. C57BL/6 or TLR4 KO mice were injected intradermally into the ear with AF488^+^ Nb. Uptake of AF488 and expression of CD86 and the IFN-I-dependent marker BST2 were examined in ear draining lymph node (dLN) DCs by flow cytometry 48 h after Nb injection. CD11b^+^ DCs were CD11c^+^ MHCII^hi^ CD11b^+^ CD326^−^ CD103^−^. Triple Negative (TN) DCs were CD11c^+^ MHCII^hi^ CD11b^−^ CD326^−^ CD103^−^. Each dot corresponds to one mouse. Data are from one of at least two repeat experiments that gave similar results. **(A)** AF488 uptake and BST2 expression on subsets of migratory DCs in dLN of C57BL/6 (WT) or TLR4 KO mice after injection of 600 AF488^+^ Nb. **(B)** BST2 expression on subsets of migratory DCs in the dLN of C57BL/6 mice after co-injection of 300 Low-Endotoxin (LE, < 5 Endotoxin Units/mL) AF488^+^ Nb with or without 10,000 formalin-fixed *E coli*. **(C)** AF488 uptake and BST2 or CD86 expression on subsets of migratory DCs in the dLN of C57BL/6 mice after injection of 600 AF488^+^ Nb with or without DNase I treatment. Bar graphs show mean ± SEM. Statistical analyses used the ANOVA with Tukey’s multiple comparisons test. Ns: not significant; *p < 0.05; **p < 0.01; ***p < 0.001.

Injection of AF488^+^ Nb is followed by the migration of AF488^+^ skin DCs from the skin to the dLN. AF488^+^ DCs in dLN peak in number at day 2 p.i. and mostly comprise the CD11b^+^ and CD326^−^CD103^−^CD11b^−^, or Triple negative (TN), DC subsets. Work from our group has shown that these two subsets of migratory DCs express an IFN-I signature that can be revealed by their expression of BST2 (11). We compared expression of BST2 on CD11b^+^ and TN DCs from WT or TLR4 KO mice, and found that, despite comparable AF488 uptake, BST2 was not upregulated in AF488^+^ DCs from TLR4 KO mice (Figure 5A). This observation is consistent with the lack of detectable IFN-I transcripts in TLR4 KO mice (Figure 4C).

Infection with live Nb also involves exposure to the microorganisms naturally associated with these helminths. Indeed Gr1^+^ cells (including, but not limited to, neutrophils, inflammatory monocytes, and pDCs), have been shown to be determinant in controlling bacterial proliferation and the survival of mice infected with Nb, thereby enabling the development of a canonical Th2 response instead of a Th1 response (27).

We investigated whether contamination by Gram-negative fecal bacteria was triggering the IFN-I response to Nb. Antibiotic-sterilized Nb preparations (sNb) (27) induced similar expression of BST2 and of the maturation marker CD86 on AF488^+^ CD11b^+^ and TN DCs infiltrating the ear dLN at day 2 p.i. (Figure S4A in Supplementary Material).

Injecting limiting numbers of an Nb preparation that had been extensively washed to lower endotoxin content (LE-Nb, <5 EU/mL versus Nb, >40 EU/mL) still induced significant BST2 upregulation on AF488^+^ DCs in dLN (Figure 5B). Co-injection of 10,000 *E. coli* together with LE-Nb enhanced the IFN-I signature of AF488^+^ DCs (Figure 5B). These results show that TLR4 ligands from helminths and/or their associated microorganisms allow the development of an IFN-I signature on migratory DCs in skin dLN.

As Nb injection induced NETosis and IFN-I secretion, we investigated whether NETs could mediate Nb-induced IFN-I expression. Regular administrations of high doses of DNase I are reported to digest NETs *in vivo* (33). Here, i.d. injection of 2,000 U DNase I together with Nb inhibited the formation of NETs at 2 h p.i., as assessed by microscopy (data not shown). However, regular injections of DNase I (i.d. and then i.p. every 12 h) was unable to reduce BST2 expression on AF488^+^ DCs in dLN at 24 h and 48 h after Nb injection (Figure 5C and data not shown). Neutrophil depletion using anti-Ly6G or anti-Gr1 antibody treatment was also unable to prevent Nb-induced BST2 expression on AF488^+^ DCs in dLN (Figure S5A,B in Supplementary Material). These results show that the IFN-I signature on dLN DCs from Nb-injected mice was not uniquely dependent on neutrophils or extracellular DNA.

Together, these data suggest that Nb and its associated TLR4 ligands induce the expression of IFN-I in the skin (7, 54–56), and an IFN-I signature on antigen-bearing migratory DCs in the dLN (11).

### TLR4 Signaling Enhances the Development of Th2 Responses to Nb

**Figure 6:**
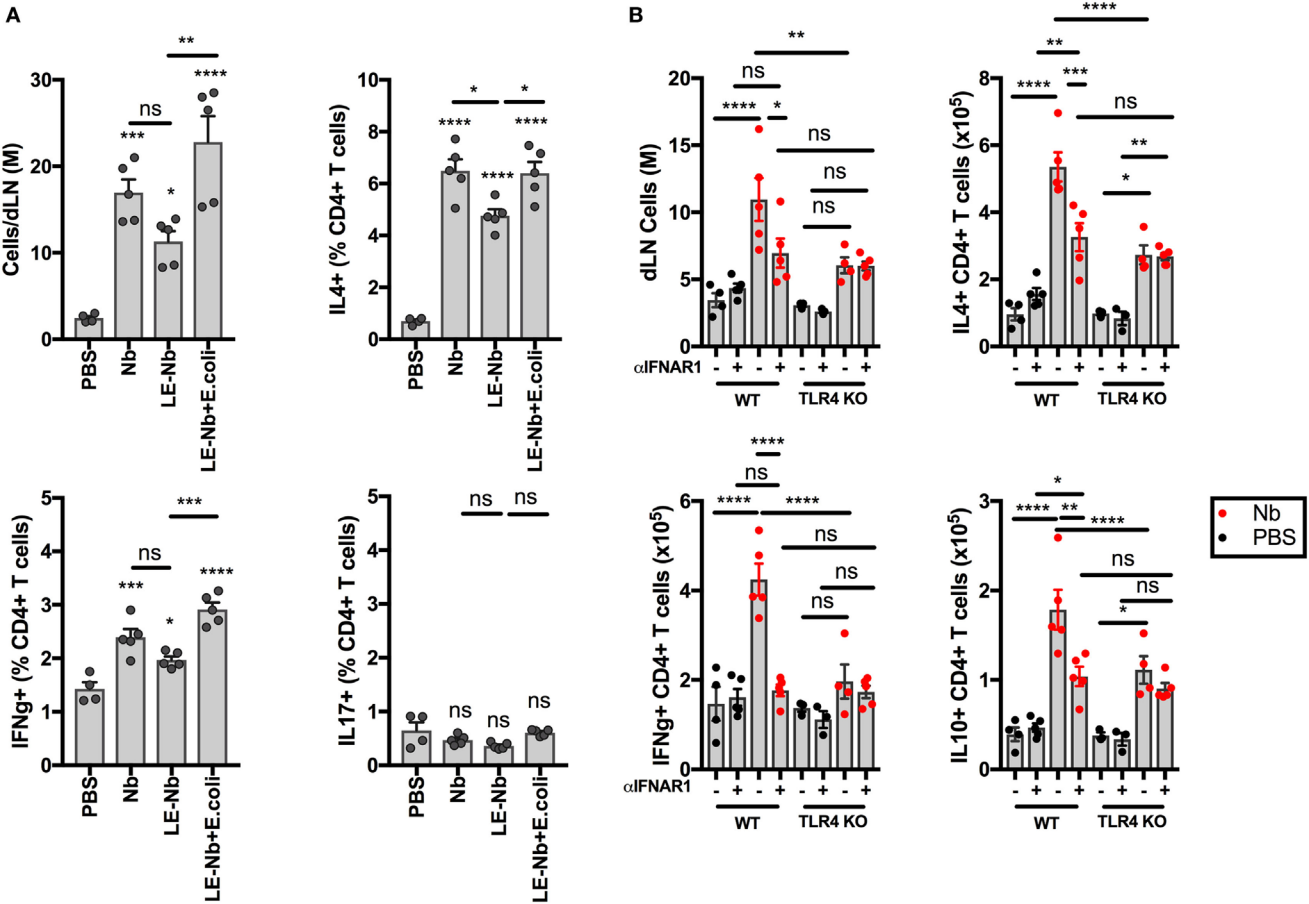
Toll-like receptor 4 (TLR4) enhances the development of CD4^+^IL4^+^ T cells after *Nippostrongylus brasiliensis* (Nb) injection. C57BL/6 or TLR4 KO mice were injected intradermally into the ear with 150 Nb from different preparations. T cell responses were measured in the draining lymph node by intracellular cytokine staining and flow cytometry 7 days after Nb injection. Each dot corresponds to one mouse. Data are from one of at least two repeat experiments that gave similar results. **(A)** T cell cytokine response in C57BL/6 mice injected with Nb, or Low Endotoxin (LE, <5 Endotoxin U/mL) Nb, or Low Endotoxin Nb preparations (LE-Nb) plus 10,000 formalin-fixed *E coli*. **(B)** T cell cytokine response in C57BL/6 (WT) and TLR4 KO mice injected with Nb together with IFNAR1-blocking antibodies or isotype control. Bar graphs show mean ± SEM. Statistical analyses used the ANOVA with Tukey’s multiple comparisons test. Ns: not significant; *p < 0.05; **p < 0.01; ***p < 0.001; ****p < 0.0001.

As shown in Figure 5, TLR4 signaling is necessary for Nb-induced BST2 expression on AF488^+^ DCs in dLN. We have previously shown that Th2 immune responses to Nb are reduced by blocking IFN-I signaling (11). We, therefore, assessed the ability of Nb preparations with different endotoxin content to induce T cell responses. As shown in Figure 6A, reducing the endotoxin content of Nb preparations decreased dLN cellularity and IL4^+^ and IFNγ^+^ T cell responses. Adding 10,000 *E. coli* to “Low-Endotoxin” Nb preparations was able to rescue the percentages and numbers of both IL4^+^ and IFNγ^+^ CD4^+^ T cells (Figure 6A). IL17A^+^ CD4^+^ T cells were not detected in significant numbers after Nb injection thus the effect of TLR4 signaling could not be assessed in this population.

We then assessed the impact of TLR4 deficiency on the contribution of IFN-I signaling to the T cell response. As shown in Figure 6B, TLR4 KO mice showed lower dLN cellularity and lower cytokine responses to Nb injection. In addition, treatment with IFNAR1-blocking antibodies was unable to decrease IL4^+^ T cell responses in TLR4 KO hosts, which is consistent with the lack of IFN-I production in these mice. A similar effect was seen also on IL10^+^ CD4^+^ T cells. IFNγ^+^ CD4^+^ T cell responses were essentially ablated by blocking IFN-I signaling and by TLR4 deficiency. Of note, a small population of IL4^+^ IL10^+^ CD4^+^ T cells was induced after Nb immunization, but was independent of IFN-I signaling or TLR4 expression (13.47 ± 2.47% of IL4^+^ CD4^+^ T cells, data not shown).

These results show that TLR4 ligands can amplify Nb-induced T cell responses through an IFN-I-dependent mechanism. The role of TLR4 and IFN-I signaling appears to be in enhancing the magnitude of the T cell immune response to Nb, rather than specifically skewing the response toward Th2.

## Discussion

In this paper, we show that the detection of Nb and/or its associated microorganisms through TLR4 allows a transient secretion of IFN-I in the skin. Preventing TLR4 signaling ablated the upregulation of IFN-I-induced markers on DCs and dampened the development of IFNγ, IL10, and IL4-secreting T cells, suggesting that TLR4 and IFNAR signaling are important but non-specific amplifiers of adaptive immune responses to Nb. We also show that Nb induces a potent recruitment and activation of neutrophils in the dermis and the formation of NETs. However, we were unable to demonstrate an effect of neutrophils or NETs on the number of Nb^+^ DCs in dLN, or on their expression of the IFN-I-induced marker BST2 and the activation marker CD86, suggesting that NET formation does not drive immune activation in response to Nb.

Neutrophils are the main cell type recruited early to the site of non-viable Nb injection. We show that neutrophils interact with AF488^+^ Nb in the skin, taking up a substantial quantity of the fluorescent dyes associated with it (Figure 1). NETs have been associated with the secretion of IFN-I in different models, and have been recently implicated in mediating the exacerbation of an asthmatic Th2 response induced by rhinovirus infection (23, 24, 34, 53). Unexpectedly, we could not find any impact of neutrophil depletion or NET digestion on the development of the IFN-I signature on antigen-bearing dLN DCs in our model. Indeed, Gr1^+^ cells, including neutrophils, have not been reported to contribute to the development of primary adaptive immune responses to sterile preparations of live Nb (27). However, neutrophils have been shown to improve memory protective responses to Nb through their control of alternative activation of macrophages in the lung (28). As NETosis is preferentially induced by the sensing of large pathogens (39), it could represent a mechanism of helminth trapping and killing (57). As antibodies and complement are important for neutrophil activation during NETosis (40), it appears to be worthwhile investigating whether the NETosis process contributes to antihelminthic protective immunity during secondary responses.

We show that endotoxins or endotoxin-containing bacteria, which are both commonly present in the helminth natural environment, can increase the Th2 response to Nb by inducing a quick and transient expression of IFN-I in the skin. Antibiotic sterilization of Nb preparations did not affect BST2 expression on AF488^+^ DCs in dLN, revealing that bacterial proliferation or secretions *in vivo* were not important for this mechanism (Figure S4A in Supplementary Material). Adding extra washing steps during Nb preparation to diminish their potential endotoxin content was sufficient to decrease the development of an IFN-I signature on AF488^+^ dLN DCs and the Th2 response to the worms. These effects could be reversed by the addition of a small number of *E. coli* at the time of Nb injection. In addition, the SN from Nb preparations could enhance the IFN-I signature on primary BM cells as much as Nb themselves (Figure 3C). Therefore, Gram-negative bacteria or endotoxins have a potential role in shaping DC activation after helminth injection, and the development of antihelminthic Th2 responses (Figures 5B and 6A). In this regard, it is nonetheless important to note that our efforts to completely eliminate the TLR4-dependent activity from Nb preparations were overall unsuccessful, suggesting the possibility that selected Nb components may be able to directly engage TLR4 and initiate signaling as was described for HDM DerP1 (58). Indeed, various helminths are reported to express glycans, glycolipids, or proteinases whose products are able to activate TLR4 (7, 54, 56, 59).

We previously showed that TLR4 deficiency had no effect on the induction of Th2 responses to Nb in IL4-reporter G4 mice (29). The Th2 immune response to Nb is dose-dependent, and using a “saturating” dose of Nb, such as 600 L3 larvae, prevented us from observing significant differences at that time. In this context, if the development of a Th2 immune response is the result of the integration of diverse redundant signals, one might not observe the contribution of each of these signals if the system is saturated by an optimal dose of stimulus. Indeed, IL25, IL33, and TSLP can each contribute redundantly to the optimal Th2 immune response in various organs and contexts, possibly compensating for the effects of TLR4-dependent IFN-I secretion (5). Here, we were able to observe a TLR4 dependency on the development of a Th2 immune response to a suboptimal dose of Nb (Figure 6B).

Our data suggest that TLR4, which is not expressed on conventional DC, enables the sensing of Nb and its associated microorganisms as a danger signal to induce IFN-I expression and indirectly enhance DC activation, maturation, and/or functional abilities (16, 60–62). The secretion of IFN-I induced by Nb was completely abrogated in TLR4 KO mice. As a result, we could not observe any effect of IFNAR blockade on the Th2 response to Nb in TLR4 KO mice. More importantly, TLR4 and its coreceptor CD14 are known to be mainly expressed by monocyte and macrophage populations. As neutrophils and monocytes were found to be the main cell types interacting with Nb early in the skin, and as neutrophils were not found to be critical in mediating Nb-induced IFN-I secretion, we can speculate that Nb sensing by monocyte and/or macrophage populations is involved in this process. Indeed, LPS recognition through TLR4 expression by macrophages is a well-known stimulus of TRIF-dependent *Ifnb* expression (47). However, we cannot exclude the possibility that other cells such as keratinocytes (63), or TLR4-mediated IFN-I independent effects, might also contribute to the development of Th2 responses. Indeed, in HDM-mediated asthma models, TLR4 expression by airway epithelial cells has been shown to contribute in a MyD88-dependent but TRIF-independent fashion to the development of an IL1-, alarmin-, and GM-CSF-mediated DC activation to allow the development of a pathogenic Th2 response (64–66). Interestingly, the expression of IRF3 by DCs was found to be critical for their optimal maturation, and for the development of asthma in similar models. However, no role was found for TRIF or IFN-I signaling through IFNAR2 blockade in those studies (67). Very recently, Webb et al reported that IFN-I signaling was indeed important for DC activation, maturation, and their induction of Th2 responses by both HDM and *Schistosoma mansoni* (12). As HDM and *S. mansoni* are both known to activate TLR4, the TLR4–IFN-I axis might be an important mechanism enhancing DC maturation and Th2 responses in various pathologies, from helminthiases to allergic diseases.

The life cycle of many parasitic helminths involves several distinct phases where helminths feed on bacteria during their free-living phase in the soil, and then spend most of their parasitic phase in the gut while infecting their host. It is difficult to conceive how a natural helminth infection could take place without involving barrier disruption of the host and a small-scale invasion by its surrounding microorganisms. Gram-negative bacteria such as *Wolbachia* can be associated with helminths, including most human-infecting filarial nematodes, in symbiotic (or obligatory symbiotic) relationships. *Wolbachia*-derived endotoxins have been shown to strongly affect the inflammatory response to *Brugia malayi* nematodes through TLR4 (68). Our results here suggest that, if DCs integrate signals from helminths and from epithelial barrier disruption to shape a Th2 immune response, the detection of the endotoxins originating from helminth-associated microflora could also participate *via* a TLR4 and IFN-I-dependent mechanism.

Our work strongly suggests that, if a limited activation of antibacterial signaling pathways is likely to occur in a natural helminth infection, it might contribute to, and not inhibit, the development of antihelminthic Th2 responses.

## Ethics Statement

All experimental protocols were approved by the Victoria University of Wellington Animal Ethics Committee (Permit 2014R17M) and performed according to Institutional guidelines.

## Author Contributions

CP carried out experiments, analyzed the data and wrote the manuscript. S-CT generated Nb stocks and carried out experiments. AS and JD designed, optimized, and analyzed confocal microscopy experiments. RW and OL carried out RT-qPCR time courses. CR provided essential reagents and experimental advice. LC provided conceptual insights and data on the model. GLG and FR provided conceptual insights and FR supervised the project. All Authors provided feedback on the manuscript.

## Conflict of Interest Statement

The authors declare that the research was conducted in the absence of any commercial or financial relationships that could be construed as a potential conflict of interest.
