# Supplementary material for: Toll-Like Receptor 4, but Not Neutrophil Extracellular Traps, Promote IFN Type I Expression to Enhance Th2 Responses to Nippostrongylus brasiliensis
Source: Front Immunol. 2017 Nov 16;8:1575. doi: 10.3389/fimmu.2017.01575 (PMC5696323; doi:10.3389/fimmu.2017.01575)
Supplement: Supplementary file 1 [file Data_Sheet_1.pdf]

Pellefigues et al:

TLR4, but not Neutrophil extracellular traps, promote IFN type I expression to enhance Th2 responses to *Nippostrongylus brasiliensis*

Frontiers in Immunology 2017

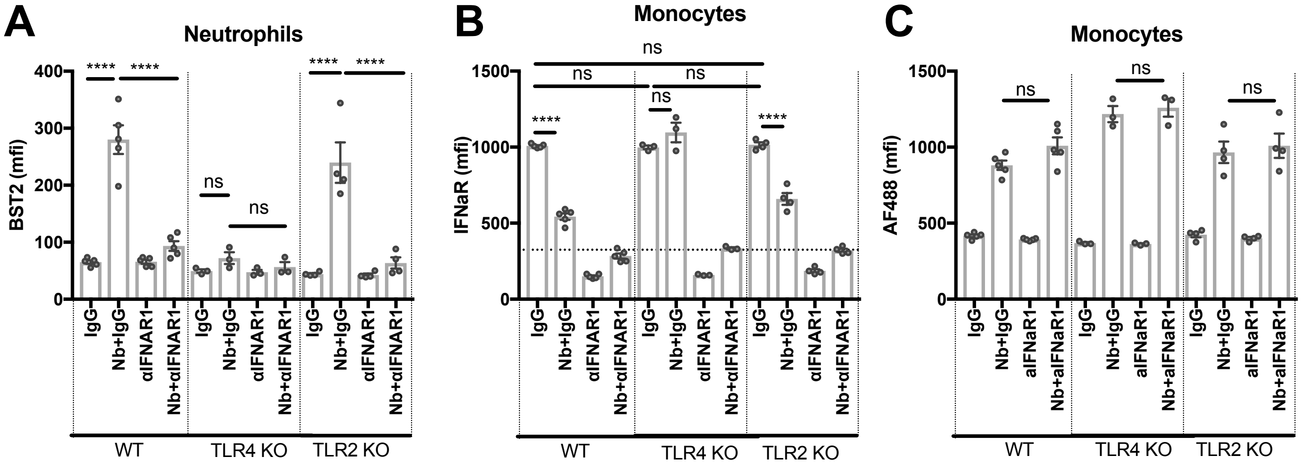

__**Figure S1:**__ Nb and *E. coli* induce an IFN-I signature on primary bone marrow cells in culture. Bone marrow (BM) cells were harvested from C57BL/6 mice or the indicated strains, stimulated for 24h by co-culture with Nb and / or *E. coli*, and analyzed by flow cytometry. Bar graphs show mean±SEM, each dot corresponds to one mouse. (**A**,**B**,**C**) Primary BM cultures from the indicated mouse strains were stimulated with AF488+ Nb, with or without anti-IFNAR1 antibodies or isotype control. Expression of (A) BST2, (B) IFNAR1, and (C) uptake of AF488 by neutrophils ($CD11b^+$ $Ly6G^+$) or monocytes ($CD11b^{hi}$ $Ly6G^-$ $Ly6C^+$ $CD64^+$) is shown. Data are from one of two independent experiments that gave similar results. Statistics were performed using one way ANOVA with Tukey's multiple comparisons test. Ns: not significant; ****: p<0.0001.

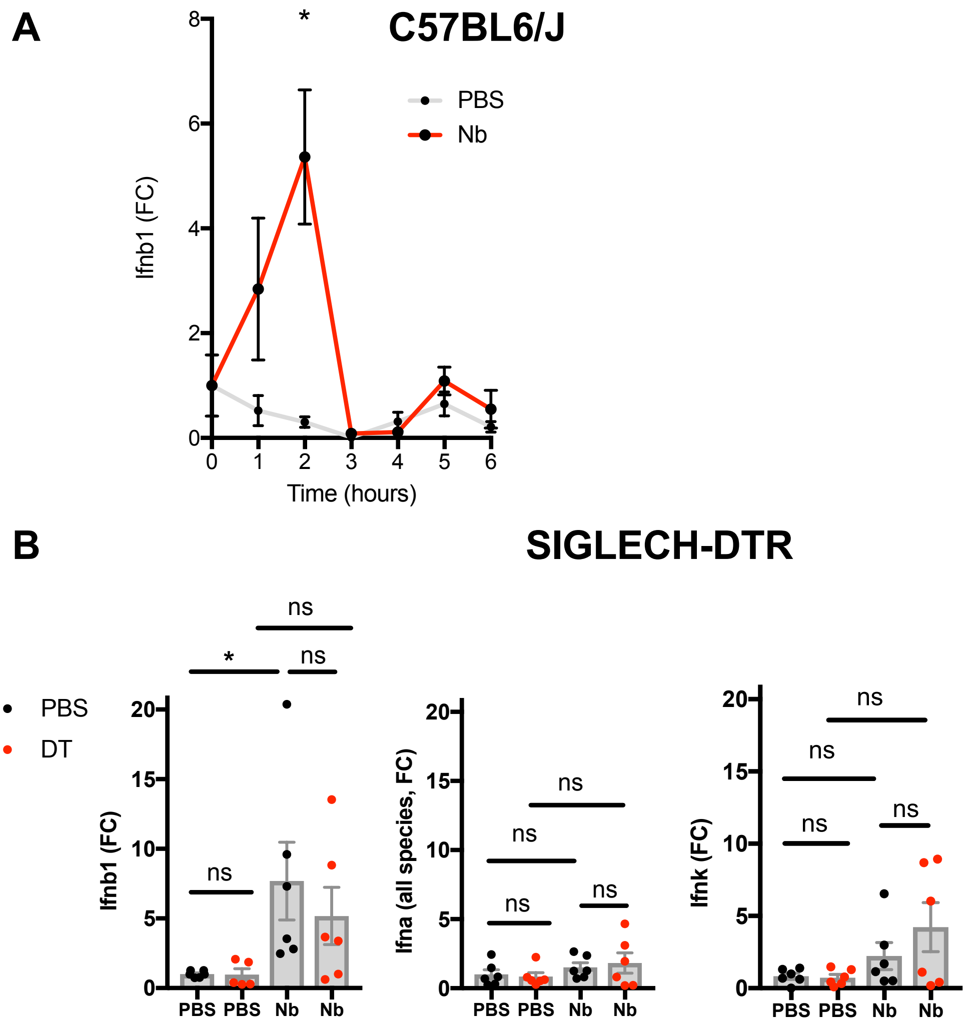

<u>Figure S2</u>: The expression of IFN-I transcripts in skin is transient and does not require plasmacytoid DCs. C57BL/6 or SiglecH-DTR mice were injected with 600 Nb or PBS in ear skin, and treated as indicated. *Ifnb1* transcripts were quantified by RT-qPCR at different time points. FC: fold-change. (**A**) Time-course of Ifnb1 transcript expression in the ear skin of C57BL/6 mice (n=3-5); means±SEM are shown. This experiment was carried out once. (**B**) Expression of Ifnb1, Ifna (all species) or Ifnk in ear skin of SiglecH mice that were depleted of plasmacytoid DCs by DT treatment, or treated with the same volume of PBS as control, before injection with Nb. Transcript levels in ear skin were assessed 2h after Nb intradermal injection. Data are pooled from two independent experiments with 3 mice/group; each symbol corresponds to one mouse. Bar graphs show mean±SEM. Statistics were performed using the Mann-Whitney test (A) or a Kruskal-Wallis test with Dunn's multiple comparison. (B) Ns: not significant; *: $p<0.05$.

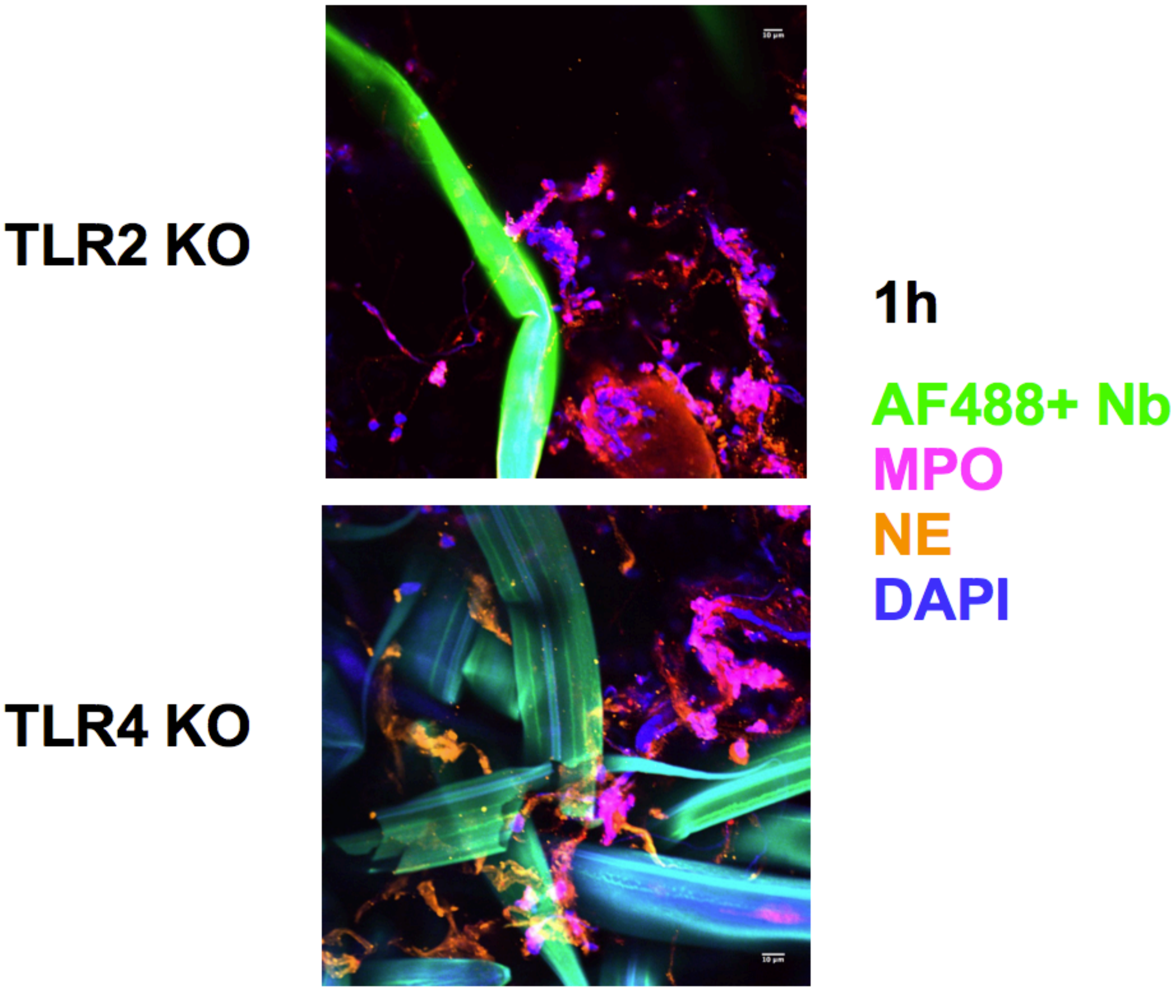

<u>Figure S3:</u> Injection of AF488^{+} Nb can induce NETosis in TLR2 KO and TLR4 KO hosts. TLR2 KO and TLR4 KO mice were injected intradermally into the ear with 150 AF488^{+} Nb. Whole-mount ear dermis was analyzed by immunofluorescence and confocal microscopy 24h after Nb injection. Z-stack images are representative of at least two independent experiments. AF488^{+} Nb (Green) is detected in the dermis and is surrounded by DAPI^{+} DNA (Blue). Myeloperoxydase (MPO, Pink) and Neutrophil elastase (NE, Orange) were detected extracellularly and were associated with extracellular DAPI staining.

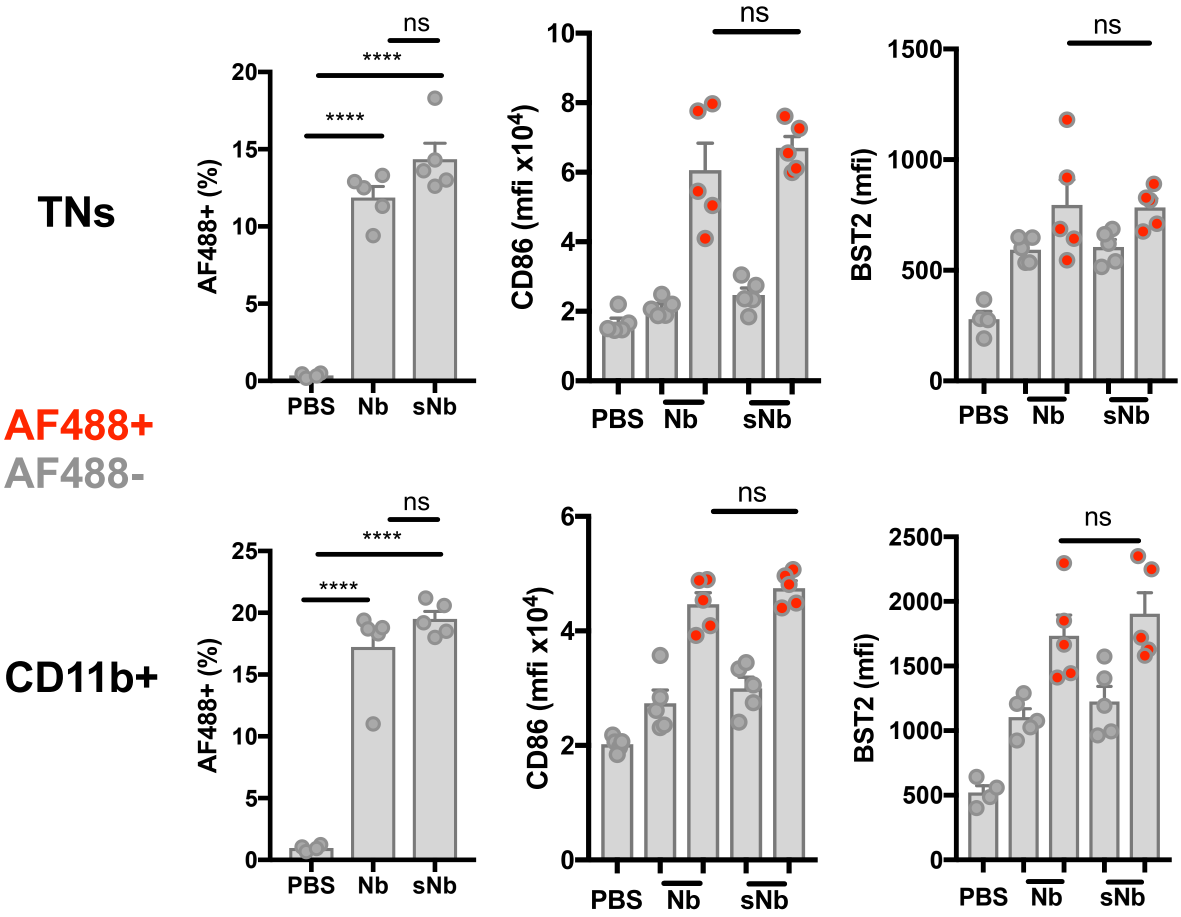

<u>**Figure S4:**</u> Nb sterilization does not prevent the development of an IFN-I signature on AF488$^+$ DCs. C57BL/6 mice were injected with PBS, 600 AF488$^+$ Nb (Nb), or 600 antibiotic-sterilized AF488$^+$ Nb (sNb) in the ear dermis. Forty-eight hours later, migratory DCs in draining LNs were examined by flow cytometry for uptake of AF488, and for expression of the costimulatory molecule CD86 and the IFN-I-induced marker BST2. CD11b$^+$ DCs were CD11c$^+$ MHCII^{hi} CD11b$^+$ CD326$^-$ CD103$^-$. Triple Negative (TN) DCs were CD11c$^+$ MHCII^{hi} CD11b$^-$ CD326$^-$ CD103$^-$. Each dot corresponds to one mouse. Data are from one of at least 2 repeat experiments that gave similar results. Bar graphs show mean ± SEM. The statistical significance of differences between groups was evaluated using an ANOVA test with Tukey's multiple comparison. Ns: not significant; ****: p< 0.0001.

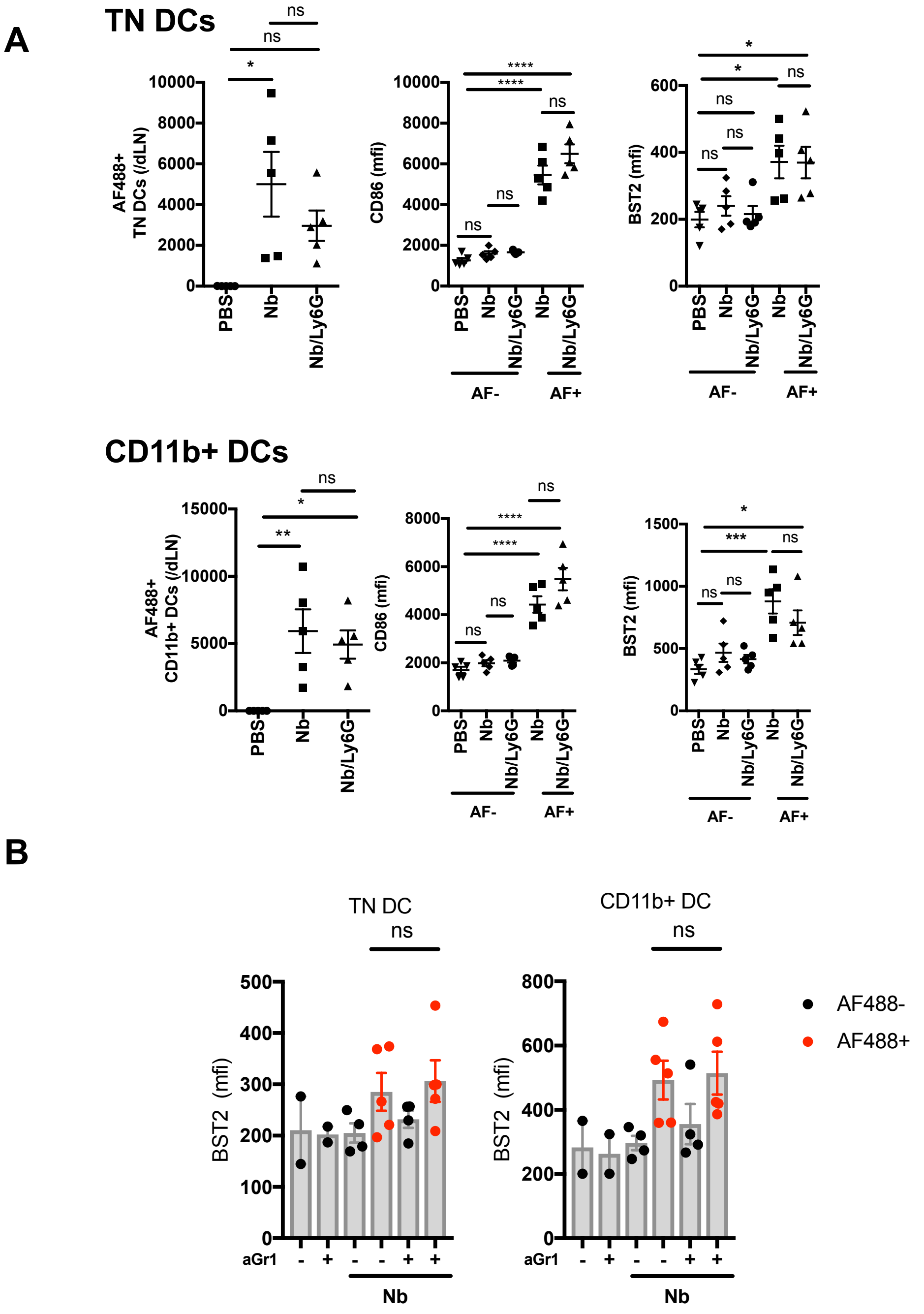

Figure S5: Neutrophil depletion does not affect the expression of BST2 on migratory DCs in the dLN of Nb-injected mice. C57BL/6 mice were depleted of neutrophils by antibody treatment and injected with 600 AF488$^+$ Nb in the ear dermis. Forty-eight hours after Nb injection, migratory DCs in draining LN were examined by flow cytometry for uptake of AF488, and for expression of the costimulatory molecule CD86 and the IFN-I-induced marker BST2. CD11b$^+$ DCs were CD11c$^+$ MHCII^{hi} CD11b$^+$ CD326$^-$ CD103$^-$. Triple Negative (TN) DCs were CD11c$^+$ MHCII^{hi} CD11b$^-$ CD326$^-$ CD103$^-$. Graphs show mean ± SEM, each dot corresponds to one mouse. (**A**) Neutrophils were depleted by treatment with anti-Ly6G antibodies. Data are from one of at least 2 repeat experiments that gave similar results. (**B**) Neutrophils were depleted by treatment with anti-Gr1 antibodies. This experiment was carried out once. The statistical significance of differences between groups was evaluated using a one-way ANOVA. Ns: not significant; *: p<0.05; **: p<0.01; ***: p<0.001.
